# Antiretroviral-Related Adipocyte Dysfunction and Lipodystrophy in HIV-Infected Patients: Alteration of the PPAR*γ*-Dependent Pathways

**DOI:** 10.1155/2009/507141

**Published:** 2008-12-30

**Authors:** Martine Caron, Corinne Vigouroux, Jean-Philippe Bastard, Jacqueline Capeau

**Affiliations:** ^1^Institut national de la santé et de la recherche médicale (Inserm), UMRS 893, 75012 Paris, France; ^2^Faculté de Médecine, Université Pierre et Marie Curie (UPMC-Paris 6), 75012 Paris, France; ^3^Assistance Publique - Hôpitaux de Paris (AP-HP), Hôpital Tenon, Service de Biochimie et Hormonologie, 75020 Paris, France

## Abstract

Lipodystrophy and metabolic alterations are major complications of antiretroviral therapy in HIV-infected patients. In vitro studies using cultured murine and human adipocytes revealed that some protease inhibitors (PIs) and nucleoside reverse transcriptase inhibitors (NRTIs) were implicated to a different extent in adipose cell dysfunction and that a chronic incubation with some PIs decreased mRNA and protein expression of PPAR*γ*. Defective lamin A maturation linked to PI inhibitory activity could impede the nuclear translocation of SREBP1c, therefore, reducing PPAR*γ* expression. Adipose cell function was partially restored by the PPAR*γ* agonists, thiazolidinediones. Adverse effects of PIs and NRTIs have also been reported in macrophages, a cell type that coexists with, and modulates, adipocyte function in fat tissue. In HIV-infected patients under ART, a decreased expression of PPAR*γ* and of PPAR*γ*-related genes was observed in adipose tissue, these anomalies being more severe in patients with ART-induced lipoatrophy. Altered PPAR*γ* expression was reversed in patients stopping PIs. Treatment of patients with agonists of PPAR*γ* could improve, at least partially, the subcutaneous lipoatrophy. These data indicate that decreased PPAR*γ* expression and PPAR*γ*-related function, resulting from ART-induced adipose tissue toxicity, play a central role in HIV-related lipoatrophy and metabolic consequences.

## 1. Introduction

HIV-associated lipodystrophy (LD) is a disorder characterized by a selective damage of the adipose tissue resulting in part from antiretroviral drugs [1, 2]. The LD syndrome includes progressive subcutaneous fat loss and/or central fat accumulation along with dyslipidemia, glucose alterations, and insulin resistance, altogether generating cardiovascular dysfunctions [3, 4]. Recent studies have hypothesized that HIV itself could play a role in the LD phenotype (see Giralt et al. [5]). However, the risk of developing fat tissue redistribution has been related in priority to the antiretroviral treatment (ART) and mainly to the use of two classes of drugs, protease inhibitors (PIs) and nucleoside reverse transcriptase inhibitors (NRTIs) [6–8]. Lipoatrophy in the face and extremities has been linked repeatedly to the use of stavudine (and to a lesser extend zidovudine) among NRTIs [7, 9, 10] and increases with long-term exposure [11]. PIs have been mainly associated with central fat accumulation along with insulin resistance. However, nelfinavir or indinavir can independently decrease limb fat level in patients cotreated with NRTIs [7, 12]. Peripheral fat loss and central fat accumulation can occur simultaneously, though lipoatrophy may emerge as the more dominant feature on prolonged treatments [12, 13]. Recently, a role for the nonnucleoside analog efavirenz in lipoatrophy has been reported but needs to be confirmed [14].

The pathogenesis of adipose cell dysfunction includes the mitochondrial toxicity of NRTIs [15–19] and the adverse effects of PIs and NRTIs on the adipocyte differentiation status [17, 20–26], insulin sensitivity [27, 28], survival [17, 18, 23, 29], ability to secrete a variety of adipokines [30–33], and longevity [19, 34]. The oxidative stress induced by both PIs and NRTIs at the fat cell level [19, 28, 33–35] probably plays a major role in the setup of lipodystrophy.

Severe adipose tissue alterations have been reported in HIV-infected patients with ART-related lipodystrophy. Lipoatrophic adipose tissue biopsies present major histological alterations with decreased and heterogeneous size of adipocytes, increased fibrosis, altered mitochondria, and macrophage infiltration [1, 2, 36–38], consistent with a profound remodeling of subcutaneous fat tissue. The presence of isolated fat droplets, macrophages, and apoptotic cells in the enlarged vascular stroma argues for a progressive destruction of subcutaneous adipocytes [1, 2, 29, 37, 39, 40].

PPAR*γ* is expressed in priority in adipocytes. It is also expressed in different other cell types including macrophages and regulates genes associated with growth, differentiation, insulin sensitivity, inflammation, and immunity [41–46] (see [5]). PPAR*γ* plays an essential role in the development and normal function of white adipocytes, where it mediates part of the regulatory effect of dietary fatty acids on gene expression [43, 47], regulates the differentiation program [48] and insulin sensitivity [45]. PPAR*γ* also controls the production and secretion of adipokines such as leptin and adiponectin, which are important mediators of insulin action in peripheral tissues [42]. In brown adipocytes, PPAR*γ* also controls the adipogenic program and the switch from white to brown adipocytes [49]. In macrophages, PPAR*γ* controls alternative activation and improves insulin resistance [50]. It plays an important role in macrophage inflammation and cholesterol homeostasis and inhibits the production of proinflammatory cytokines through inhibition of the NF*κ*B and AP-1 pathways [48, 51–54].

Loss-of-function or dominant-negative mutations in the PPARG gene in humans (see [5]), and genetically-induced PPAR*γ* deficiency in mice [55, 56] are responsible for lipodystrophic syndromes with insulin resistance, showing the primarily involvement of PPAR*γ* defects in adipose tissue development and metabolic roles. Alternatively, other causes of adipocyte differentiation defects lead to a secondary decreased PPAR*γ* expression and/or function, that further contribute to adipose tissue dysfunction, as shown in vivo in murine models [57] or in vitro [58–60].

In that setting, the implication of PPAR*γ* in the ART effect has been demonstrated both in vitro, in cultured adipocytes and macrophages, and ex vivo, in adipose tissue samples from patients, and has been confirmed by the beneficial effects, at least partial, of the PPAR*γ* agonists, thiazolidinediones. PPAR*γ* defects, although probably secondary to the multiple deleterious consequences of ART on adipose tissue, play a central role in ART-related lipodystrophy and metabolic alterations.

## 2. Effects of ART on PPAR*γ* Expression and Signaling in Cultured Adipocytes

PPAR*γ* contributes to the setup of the differentiation program and to insulin sensitivity. PIs and NRTIs, the two major classes of antiretrovirals associated with lipodystrophy in HIV-infected patients, may interfere at several steps of PPAR*γ* signaling in adipose cells, such as differentiation, insulin action, oxidative stress, inflammation, and mitochondrial function.

A number of studies have clearly shown that the first generation PIs, indinavir, nelfinavir, and ritonavir, used at concentrations comparable to their Cmax in patients' serum or at suprapharmacological concentrations, impaired adipocyte differentiation [20, 21, 23, 25, 26, 32, 61–67]. They were also shown to induce insulin resistance [21, 23, 27, 33, 62, 67–70] in murine and human cultured adipocytes. This was associated with a reduced protein and mRNA expression of PPAR*γ* in both murine [20, 21, 25, 26, 64] and human adipocytes [24, 66, 71, 72]. Interestingly, decreased PPAR*γ* expression was also observed in mature adipocytes chronically incubated with PIs, consistent with PI-induced adipose cell dedifferentiation.

Most PIs (nelfinavir, indinavir, saquinavir, ritonavir, and amprenavir) were shown to acutely inhibit insulin activation of glucose uptake in cultured adipocytes, via a direct inhibition of the glucose transporter Glut4 [73]. Indinavir and nelfinavir also altered the activation of proximal steps in insulin signaling as revealed by decreased phosphorylation of extracellular-regulated kinase (ERK) 1/2 and Akt/protein kinase B. Accordingly, distal events in insulin signaling pathways, glucose transport, and lipogenesis were also affected [21, 30, 74]. Regarding PPAR*γ*, cell imaging studies revealed that indinavir and nelfinavir but not amprenavir severely decreased nuclear expression of PPAR*γ* [21], indicating for the first time that the transcriptional activity of PPAR*γ* may be defective in PI-treated cells. The beneficial effect of rosiglitazone [21, 23, 32] confirmed the implication of PPAR*γ* in PI action, and indicated that PIs act upstream of PPAR*γ* in its signaling cascade to alter adipocyte differentiation and insulin sensitivity. Recent data of our laboratory further support the implication of PPAR*γ* in PI action by showing that two angiotensin II-receptor blockers (telmisartan and irbesartan), that display partial PPAR*γ* agonist activity [75], prevented the PI effects on lipid accumulation and insulin response in murine and human adipocytes (Boccara F. et al., unpublished results).

The effect of ritonavir on insulin signaling has been particularly studied since this commonly prescribed PI is associated with dyslipidemia and metabolic disorders in HIV-infected patients [67, 76, 77]. Ritonavir induced insulin resistance in cultured adipocytes [24, 32, 64]. Another study reported that ritonavir reduced differentiation and insulin sensitivity in human preadipocytes and adipocytes but surprisingly without decreasing PPAR*γ*2 gene expression [68]. However, the protein expression and the activation of PPAR*γ* have not been evaluated in this study.

The mechanism whereby PIs alter adipose cell differentiation and insulin sensitivity is obviously complex and multifactorial. Impaired SREBP-1 nuclear penetration [21, 22] may inhibit the activation of PPAR*γ* or related adipogenic transcription factors thus leading to defective adipogenesis and insulin resistance. When going further into the mechanism of PI action, we and others demonstrated that some PIs prevented the maturation of lamin A/C [22, 34, 78], a nuclear membrane protein essential for normal nuclear membrane folding and for nuclear penetration of SREBP-1 [59, 79, 80]. Defective SREBP-1c signaling may explain the decreased differentiation and insulin resistance of PI-treated cells and the ability of PPAR*γ* agonists to overcome the PI effects on fat cell differentiation and insulin response [21].

NRTI therapy is also associated with fat tissue disease in HIV-infected patients. In murine adipose cell lines and primary cultured human adipocytes, stavudine and zidovudine, but not other NRTIs (tenofovir, abacavir, didanosine, and lamivudine), alter lipid storage [23, 31, 33, 81]. They also decrease the expression and secretion of adiponectin in cultured human and murine adipocytes [23, 32, 33, 82] and induce oxidative stress, suggesting that they could secondarily participate to the insulin resistance setup [33]. The negative effect of NRTIs on PPAR*γ* expression and signaling has been reported only in a few studies. Stavudine or zidovudine have a modest, or no effect, on adipose cell differentiation assessed by the gene expression profile of differentiating adipocytes [25] and by protein and mRNA expression of adipogenic transcription factors, among them PPAR*γ* [20, 25, 31, 32, 82]. Altered adipocyte lipid phenotype and insulin sensitivity resulting from NRTI treatment are suspected to result from their mitochondrial toxicity [15–18]. We recently reported that stavudine or zidovudine, but not other NRTIs, triggers mitochondrial oxidative stress and premature senescence in cultured fibroblasts and adipocytes [19]. Stavudine also altered in human preadipocytes [72] the expression of the PPAR*γ* coreceptor 1-alpha (PGC1-*α*) a transcriptional coactivator upregulated by thiazolidinediones which controls mitochondrial function and biogenesis, and metabolic pathways and integrates insulin signaling and mitochondrial function [83, 84]. Stavudine increased its expression together with mitochondria number [72]. Thus, conversely to PIs, in vitro, thymidine analogs have no or mild detrimental effect on PPAR*γ* function.

The non-NRTI class of antiretrovirals has not yet, as a class, been associated with long-term toxicity [7] even if efavirenz was shown in one study to be associated with lipoatrophy [14]. Very few studies report experimental in vitro findings on the effects of the non-NRTIs efavirenz or nevirapine on white adipose cell functions. Efavirenz but not nevirapine induced a delayed and moderate reduction in lipid accumulation in both murine and human cultured adipocytes, and decreased SREBP-1c and PPAR*γ* expression [85].

## 3. Effect of ART on PPAR*γ* Expression and Function in Animal Models

Ritonavir was shown to increase lipogenesis [86] and to induce insulin resistance in animal models [87]. In mouse fat tissue, it partially inhibits the function of PPAR*γ* as shown by the decreased induction of PPAR*γ* target genes by rosiglitazone [88]. Lopinavir-ritonavir but not atazanavir decreased by 25% the weight of peripheral inguinal fat in mice treated for 8 weeks, while the profound epididymal adipose tissue depot was not affected. The expressions of SREBP-1c and of its target gene fatty acid synthase were increased in the peripheral inguinal fat while that of PPAR*γ* tended to be decreased in the two depots and that of its target gene adiponectin was not modified [89]. Even if not entirely conclusive, these data are in favor of an altered expression and/or function of PPAR*γ* induced by some PIs in murine models.

## 4. Effect of ART on PPAR*γ* Expression and Function in Patients' Adipose Tissue

Studies performed on human adipose tissue samples studied ex vivo concerned, at first, healthy controls treated with ART. Mallon et al. [90] reported that a 2-week treatment with stavudine/lamivudine or zidovudine/lamivudine resulted in an increased expression of PGC1*α* and PPAR*α* and a decreased expression of PPAR*γ* without any modification in the expression of SREBP1. Altered expression of PGC1*α* was correlated with upregulation of nuclear genes involved in transcription regulation of mtRNA and oxidation of fatty acids suggesting a central role for PGC1 in nuclear response to mitochondrial dysfunction.

Several studies evaluated the expression of PGC1*α* and PPAR*γ* in adipose tissue from long-term ART treated HIV-infected patients with lipodystrophy. A decreased expression of the two factors was reported in abdominal fat from lipodystrophic patients as compared to controls [36, 37] and to non-lipodystrophic patients [91]. A decreased expression of PPAR*δ* was also found in this latter study. Accordingly, a decreased expression of the transcription factor SREBP-1 was also reported [36, 91, 92]. PPAR*γ* adipose tissue expression was found decreased in HIV-infected patients as compared to noninfected controls by Giralt et al. [5] but the major decrease was observed in naïve versus ART-treated patients, without differences between lipodystrophic and nonlipodystrophic patients, arguing for a major role for the virus itself. The expression of PGC1*α* was increased. The group of D. Nolan and S. Mallal observed that the PPAR*γ*2 mRNA level was similar in fat from treatment-naïve patients and in patients under PI or zidovudine but lower in patients under stavudine. However, noninfected controls were not evaluated in that study [93]. Interestingly, adipose tissue dysfunction appears more severe in peripheral than in abdominal subcutaneous adipose tissue, as shown by the decreased expression of PPAR*γ*, C/EBP*α*, and adiponectin in adipose tissue from thigh versus abdomen [94]. Therefore, a strong alteration in PPAR*γ* expression was found in most studies using HIV-infected patients' subcutaneous fat samples.

To examine the reversibility of adipose tissue alterations in HIV-infected patients, adipose tissue biopsies were studied before and after a 6-month interruption of ART in the Lipostop study. Adipose tissue inflammation improved markedly, with fewer infiltrating macrophages and fewer TNF*α*- and IL6-expressing cells. mRNA expression of PPAR*γ* and of markers of mitochondrial function and biogenesis (cytochrome oxidase subunit 2 and PGC1*α*) improved after PI withdrawal. In patients who stopped taking stavudine or zidovudine, adipose tissue inflammation, mitochondrial status, and SREBP-1 expression were improved [95]. Since PGC1*α* is playing a leading role in mitochondria function [84], this indicates that altered PGC1*α* and PPAR*γ* expression induced by some ART may be involved in mitochondria dysfunction observed in patients' fat [90, 95].

Decreased PPAR*γ* expression was also strongly correlated with increased expression of inflammatory cytokines such as IL-6 and TNF-*α* and decreased expression and circulatory levels of adiponectin which is involved in liver and muscle insulin sensitivity [1, 36, 37, 91, 96]. These data confirm that altered PPAR*γ* function in adipose tissue plays a role in overall insulin resistance associated with lipodystrophy, as reported in genetically-determined PPAR*γ* dysfunctions [45]. In accordance, the study from Sutinen et al. [97] reported the effects on adipose tissue of a 24-week treatment with the PPAR*γ* agonist rosiglitazone compared with placebo in HIV-infected patients with lipodystrophy. The expression of adiponectin, PPAR*γ*, and PGC1*α* significantly increased while that of IL-6 decreased. Expression of other genes involved in lipogenesis, fatty acid metabolism, or glucose transport, such as PPAR*δ*, and SREBP-1, remained unchanged. Rosiglitazone also significantly induced an increase in serum adiponectin concentration, which was inversely correlated with the changes in fasting serum insulin concentration and liver fat content. Such data have led to conduct clinical trials using thiazolidinediones to try to reverse peripheral fat loss. Even if the results obtained with rosiglitazone were disappointing (see [97]), possibly due to the ongoing presence of stavudine in the ART regimen, recent data obtained with pioglitazone are more promising and reveal, in patients not treated with stavudine, an improvement of peripheral fat [98] further supporting a role for PPAR*γ* dysfunction in lipoatrophy.

## 5. PPAR*γ* Expression and Fat Hypertrophy in HIV-Infected Patients

The lipodystrophic phenotype observed in HIV-infected patients associates, to different extent, peripheral lipoatrophy and fat hypertrophy in different fat depots. In particular, a buffalo hump has been observed in a number of patients. The group of F. Villaroya showed that buffalo humps from HIV-infected patients displayed a brown adipose tissue phenotype with both specific uncoupling protein 1 (UCP1) expression and mitochondrial dysfunctions [99]. However, there were no significant changes in the expression of other UCP genes or of that of markers of adipogenesis including PPAR*γ*, PGC1*α*, and adiponectin relative to controls. A more extensive analysis indicated that buffalo hump tissue does not express a complete brown adipocyte phenotype but rather a distorted brown-versus-white phenotype associated with enhanced proliferation [2]. In addition, buffalo humps failed to show increased expression of TNF*α* or the macrophage marker CD68 indicating the absence of a local inflammatory status. Since adipose tissue inflammation and the presence of proinflammatory cytokines has been presumed to play a role in subcutaneous fat lipoatrophy in HIV-infected patients, this absence of inflammation could explain, at least in part, the absence of fat loss observed in that depot.

The effect of antiretrovirals on brown adipocytes has been evaluated in two studies. In primary culture of differentiated murine brown adipocytes, neither the cell differentiation nor the level of PPAR*γ* was modified by the treatment with a series of NRTI including stavudine and zidovudine. By contrast, regarding the NNRTI, nevirapine increased and efavirenz decreased brown adipocyte differentiation and PPAR*γ* expression. PGC1*α* expression was not modified by the drugs except for its increase in response to stavudine and nevirapine [100]. In the T37i brown adipocyte cell-line, indinavir, stavudine, and zidovudine alone or in association impaired PPAR*γ*2 and UCP1 expression together with a strong inhibition of cell differentiation and mitochondrial functions, although the 3T3-F442A white adipocyte cell line, studied under similar conditions, was less severely affected [26]. Therefore, brown fat can also be a target of antiretrovirals. Since the presence of brown adipose tissue in normal humans has been recently reassessed [101], it would be important to further evaluate its alterations in HIV-infected patients under ART.

Increased visceral fat is also a characteristic feature of HIV-related lipodystrophy. However, samples from patients are difficult to obtain and no study, up to now, has reported specific data obtained with HIV-infected patients' visceral fat. A few studies compared the effect of antiretrovirals on adipocytes issued from subcutaneous and visceral fat from noninfected subjects but the expression of PPAR*γ* or PGC1 was not evaluated.

## 6. PPAR*γ* and Macrophages

PPAR*γ* plays an important role in macrophage function and phenotype and exerts an overall anti-inflammatory function (see [5]). Recent data have shown that adipose tissue from obese individuals presents macrophage infiltration as well as increased number of “M1” or “classically activated” macrophages. Importantly, the agonists of PPAR*γ* have been shown to alter macrophage phenotype to “M2” or an “alternatively activated” anti-inflammatory phenotype and may induce macrophage specific cell death [102]. PIs could alter PPAR*γ* in macrophages by increasing PPAR*γ* mRNA expression resulting in foam cell formation [103]. In the Lipostop study [95], we observed that stopping ART resulted in an improvement of adipose tissue function associated with a decreased number of M1 but not M2 macrophages together with an increased expression of PPAR*γ*. This can result from modified PPAR*γ* expression both in adipocytes and macrophages.

## 7. Conclusion

In vitro and in vivo data strongly suggest that altered PPAR*γ* function plays a role in HIV-related lipodystrophy as a result of a multifactorial toxicity of ART on adipose tissue. In vitro studies investigating the effect of individual antiretrovirals have clearly revealed that some PIs inhibit PPAR*γ* functions, probably at the earlier step of SREBP1c activation. Ex vivo studies of adipose tissue, both in healthy volunteers and in HIV-infected patients, confirmed these data but also point to a possible toxicity of NRTI, principally stavudine and to a lesser extent, zidovudine. Since PPAR*γ* is playing a central role in adipose tissue differentiation and function, decreased PPAR*γ* expression could be expected to be involved in the pathophysiology of lipodystrophy. Importantly, both adipocytes and macrophages present in patients' adipose tissue can be affected at the PPAR*γ* level. Adipose tissue dysfunction could induce insulin resistance and deregulate adipokine secretion with increased release of proinflammatory cytokines and decreased adiponectin, alterations which will impact on the liver and muscles.

Most studies in that setting evaluated the expression and function of PPAR*γ* and only scarce data are available for PPAR*α* and PPAR*δ*.

Using thiazolidinediones to reverse fat lipoatrophy was a logical proposition. However, trials using rosiglitazone were disappointing, in part due to the absence of discontinuation of stavudine. Pioglitazone was more promising and resulted in some recovery of limb fat further arguing for a role for PPAR*γ* in initial fat alteration.
